# Targeted heart rate control using the funny current inhibitor ivabradine to reduce morbidity in patients undergoing noncardiac surgery: study protocol for a phase 2a, triple-blind, placebo-controlled randomised trial

**DOI:** 10.1016/j.bjao.2025.100378

**Published:** 2025-02-19

**Authors:** Bernardo Bollen Pinto, Benjamin Shelley, Priyanthi Dias, Salma Begum, Florence Ennahdi-Elidrissi, Tom E.F. Abbott, Russell Hewson, Akshaykumar Patel, Kamran Khan, Rupert M. Pearse, Gareth L. Ackland, for the FUNNY trial investigators, Bernardo Bollen Pinto, Benjamin Shelley, Priyanthi Dias, Salma Begum, Florence Ennahdi-Elidrissi, Russell Hewson, Anna Wozniak, Shaun M. May, Mareena Joseph, Agustine Miguel Saavedra, Tim Martin, Onika Ottley, Ana Santos, Fatima Seidu, Stéphanie Mulin, Stéphane Luise, Isabelle Pichon, John Daniels, Béatrice Gil-Wey, Soraya Bicher, Gaël Rais, Christene Aitken, Elizabeth Boyd, Patricia Griffen, Charlene Hamilton, Kathryn Valdeavella, Rhiannon McAreavey, Phillip McCall, Alfie Lloyd, Jocelyn Barr, Julie Buckley, Anne Marie Tiah, Henrike Janssen, Lisa Kandala, Angela Fitzpatrick, Alexander Lysomirski, Ahmed Ahltobi, Ana Gutierrez del Arroyo, Tom E.F. Abbott, Akshaykumar Patel, Kamran Khan, Rupert M. Pearse, Gareth L. Ackland

**Affiliations:** 1 Department of Anaesthesiology, Pharmacology, Intensive Care and Emergency Medicine, Geneva University Hospitals, Geneva, Switzerland; 2 Department of Cardiothoracic Anaesthesia and Intensive Care, Golden Jubilee National Hospital, Clydebank, UK; 3 Anaesthesia, Perioperative Medicine and Critical Care Research Group, University of Glasgow, Glasgow, UK; 4 Faculty of Medicine and Dentistry, William Harvey Research Institute, Queen Mary University of London, London, UK

**Keywords:** autonomic nervous system, elective surgical procedures/adverse effects, heart rate, myocardial injury, postoperative complications, prospective studies, vagus nerve

## Abstract

**Background:**

Myocardial injury is strongly associated with excess morbidity and mortality after noncardiac surgery. Higher heart rate may result in perioperative myocardial injury through demand–supply mismatch. Alternatively, higher heart rates may reflect autonomic dysfunction that promotes myocardial injury independently of heart rate. The specific hyperpolarisation-activated, cyclic nucleotide-gated (HCN)-4 (funny) channel inhibitor ivabradine slows the heart rate without altering autonomic control, blood pressure, or myocardial contractility. We hypothesise that individuals with autonomic dysfunction may benefit most from ivabradine reducing heart rate control to minimise myocardial injury-associated morbidity.

**Methods:**

This triple-blind, international, multicentre, randomised, placebo-controlled, parallel group randomised trial will recruit 350 patients, aged ≥55 yr, with cardiovascular risk factors for myocardial injury during elective noncardiac surgery. To achieve the target heart rate <70 beats min^−1^ (sinus rhythm), patients will be randomly allocated in a 1:1 ratio using minimisation and will receive either ivabradine (2.5–7.5 mg) or placebo tablet twice daily, from the morning of surgery for 72 h. High-sensitivity troponin T concentrations will be measured before and up to 72 h after surgery, blinded to participants, clinicians, and investigators. The primary outcome is myocardial injury associated with morbidity within 7 days of randomisation (defined by Postoperative Morbidity Survey). Secondary outcomes include peak troponin concentrations, complications within 30 days, and mortality within 6 months of surgery. Pre-specified analyses will include resting and orthostatic heart rate plus N-terminal prohormone of brain natriuretic peptide concentrations before surgery.

**Conclusions:**

This phase 2b study will explore whether targeted heart rate control reduces morbidity after surgery, using ivabradine to selectively slow the heart rate without altering perioperative autonomic control.

**Clinical trial registration:**

ISRCTN12903789.

At least 300 million surgical operations are estimated to occur each year globally.^1^ Myocardial injury after noncardiac surgery occurs in ∼40% of the highest risk older patients with comorbidity who undergo elective major surgery.^2^ Myocardial injury is defined by prognostically significant elevations in troponin I or T using high-sensitivity troponin assays.^3^ Although the majority of these episodes that occur within 48 h of surgery are asymptomatic, myocardial injury after noncardiac surgery is independently and strongly associated with both short-term morbidity^4^^,^^5^ and longer-term complications including mortality.^6^^,^^7^

Because myocardial injury usually occurs in the absence of chest pain and electro- and/or echocardiographic changes consistent with myocardial infarction,^8^ numerous non-thrombotic mechanisms have been postulated to trigger this sentinel pathophysiological event.^9^ Supply–demand mismatch may occur as a result of haemodynamic lability^10^^,^^11^ and tachycardia,^12^^,^^13^ which increase myocardial oxygen consumption. Higher preoperative heart rates >83 beats min^−1^ are independently associated with myocardial injury.^12^^,^^13^ However, limiting potentially injurious tachycardia with beta-blockers and clonidine resulted in hypotension and ischaemic stroke.^14^ Poise-1 highlighted the hazards of using fixed dose, extended-release beta-blockers without titration or individualised dosing.^15^

Resting heart rate is chiefly determined by vagal (parasympathetic) tone.^16^ The association between perioperative organ injury and higher heart rate may therefore reflect vagal autonomic dysfunction that promotes cardiovascular injury^17^^,^^18^ and multiorgan dysfunction^19^^,^^20^ through inflammatory and cellular protective mechanisms that are independent of heart rate alone.^21^ The Measurement of Exercise Tolerance before Surgery (METS) study investigators reported that delayed heart rate recovery after exercise, an exercise-evoked measure of vagal tone, was independently associated with myocardial injury after noncardiac surgery.^22^ Additional mechanistic work suggests that dynamic perioperative changes in vagal tone are also associated with myocardial injury.^23^ However, the observational design of these studies cannot provide further insights as to whether higher heart rate *per se*, or vagal dysfunction independently of heart rate, underpins the association with higher rates of postoperative morbidity (Fig. 1).

**Fig 1:**
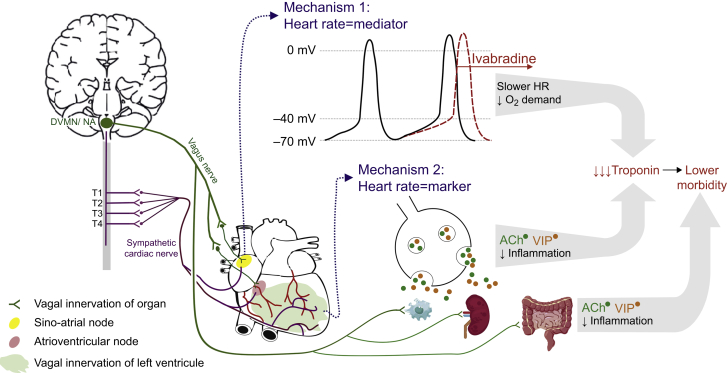
Hypothesis for *the* FUNNY RCT. If elevated heart rate (HR) is a direct mediator of myocardial injury through driving oxygen demand mismatch, the selective inhibition by ivabradine of the funny channel, located in the sino-atrial node (upper yellow area), should reduce myocardial injury and associated morbidity. If elevated heart rate is a biomarker for morbidity, its chief determinant—efferent vagal activity—is reduced. Loss of cardiac vagal function results in reduced cardioprotection (highlighted green area) and additional extracardiac mechanisms that reduce inflammation and confer organ protection in systemic inflammation and ischaemia–reperfusion injury. Cellular protection may be attributable to numerous mechanisms mediated by neurotransmitters released by the vagus nerve, acetylcholine (ACh), and vasoactive intestinal peptide (VIP), dorsal motor nucleus of the vagus nerve (DVMN), nucleus ambiguus (NA).

In contrast to beta-blockers, ivabradine is a specific I(f) current inhibitor that decreases heart rate without altering autonomic control, myocardial contractility, and blood pressure.^24^ These properties have fuelled the use of ivabradine in not only chronic heart failure but also acute cardiac care, where a recent meta-analysis showed that ivabradine effectively decreases heart rate without haemodynamic compromise.^25^ A recent pilot randomised controlled study extended these findings to the first 3 days of the perioperative period, where individualised use of ivabradine was shown to be safe and deliverable.^26^ These properties afford the opportunity to test the hypothesis that individuals with autonomic dysfunction will benefit most from ivabradine reducing heart rate control to minimise myocardial injury-associated morbidity. Ivabradine offers a pharmacological intervention to dissociate heart rate from both established and acquired autonomic dysfunction that may cause organ injury.

In the FUNNY RCT described herein, we hypothesise that targeted heart rate control with ivabradine for the first 72 h of the perioperative period will reduce early myocardial injury and subsequent morbidity within 7 days after surgery. In particular, the protective effect of targeted heart rate control may be more pronounced in participants with poor baseline cardiovascular function (high preoperative N-terminal prohormone of brain natriuretic peptide [NT-proBNP]), vagal autonomic dysfunction (higher preoperative resting heart rate, impaired orthostatic heart rate response), or both.

## Methods and analyses

The protocol was developed in accordance with Standard Protocol Items for Randomized Trials (SPIRIT) recommendations.^27^

### Study design

Multicentre, international, triple-blind, placebo-controlled, parallel group randomised controlled trial.

### Setting

Surgical services of three academic hospitals in Europe. Participant recruitment started in January 2022 and is scheduled to complete in 2025. Recruiting site eligibility criteria include having surgical services performing major elective surgery in adults, the ability to prepare and store frozen plasma/serum samples, and previous participation in interventional research.

### Participants

#### Inclusion criteria

Adults aged ≥55 yr undergoing major non-emergent surgery typically lasting ≥120 min. Participants must have at least one medical risk factor for perioperative myocardial injury (Table 1), which may include a history of hypertension (requiring antihypertensive drug) or hypertension recorded in pre-assessment clinic (BP >140 mm Hg systolic, >90 mm Hg diastolic).

**Table 1:** Medical risk factors for the FUNNY randomised controlled trial.

• Coronary artery disease
• Diabetes mellitus requiring oral hypoglycaemic agent, insulin, or both
• Congestive heart failure
• Cerebrovascular accident
• Chronic kidney disease stage 3–5 (eGFR <59 ml min^−1^ 1.73 m^−2^)
• Peripheral arterial disease
• History of hypertension (requiring antihypertensive drug) or hypertension recorded in pre-assessment clinic (BP >140 mm Hg systolic; >90 mm Hg diastolic)

#### Exclusion criteria

Participants are ineligible if they are already participating in a clinical trial of a treatment with a similar biological mechanism (including previous participation in FUNNY), are unable to provide, or refuse, informed consent, or lack capacity to do so. From a cardiovascular perspective, atrial fibrillation (persistent/chronic or paroxysmal) and prior use of ivabradine within 30 days before surgery preclude enrolment. Absolute contraindications to ivabradine are provided in appendix, including a history of hypersensitivity or allergy to ivabradine or any of its excipients. Women of childbearing potential (premenopausal female capable of becoming pregnant unless permanently sterile) cannot participate.

### Enrolment

Coordinated trial leadership at an international, national, and hospital level has ensured local engagement of surgeons, anaesthetists, and intensivists to support screening and trial delivery. Each site has experienced local investigators and research teams, informed by extensive public and patient input to the trial design. Recruitment targets are monitored and actively managed throughout the trial.

Potential participants are screened by research staff after being identified by clinical teams from pre-admission clinic lists and operating lists. Before surgery, potential participants are approached at least 24 h before their operation to discuss the aims, methods, anticipated benefits, and potential harms of the trial, and receive a patient information sheet (Supplementary material). Written informed consent is obtained from each subject on the day of surgery once surgery is confirmed to proceed. The reasons for eligible patients not participating in the trial will be recorded.

### Randomisation

Once surgery on the day is confirmed to proceed, randomisation will occur after the participant has provided informed consent. Participants are randomised in a 1:1 ratio by minimisation with a random component, with group allocation carried out using a central online service. Minimisation variables will be hospital site and surgical procedure (surgery involving the gut or all other surgery). Each participant will be allocated with 80% probability to the group that minimises between-group differences in these factors among all participants recruited to the trial to date, and to the alternative group with 20% probability. The allocation sequence is generated by an automated algorithm and is concealed to all trial investigators. The system for generating the allocation sequence will be bespoke and will be developed in-house. The system will be validated for use by the trial statistician.

### Study interventions

Usual perioperative care will proceed in each centre for participants in this trial. Blood samples will be collected and prepared for serum and plasma before surgery, 24, 48, and 72 h after surgery, between 06:00 and 10:00, before freezing at –80°C, thus maintaining blinding of the primary outcome. Measurement of high-sensitivity troponin in batched frozen samples stored for 928 days does not affect the results providing values indistinguishable from ‘on-demand’ fresh samples (including using these results for the clinical application of rapid diagnostic algorithms).^28^

Before dosing, resting heart rate and orthostatic heart rate changes from sitting to standing are recorded, which quantify cardiac vagal activity.^29^ Participants receive 0–3 tablets that are indistinguishable, being either 2.5 mg of ivabradine or placebo, based on the participant's resting heart rate within 1 h before dosing (Table 2), unless MAP is <60 mm Hg or a new-onset arrhythmia has been recorded. If multiple heart rate measurements have been recorded within 1 h before dosing, the lowest value is taken to determine the dose of the investigational medicinal product (IMP). Dosing is performed by research staff and will occur twice daily from the morning of surgery until the evening of postoperative day 2 (3 days, six administrations). The individualised variable dosage has been shown in our pilot study to achieve the target heart rate of 60–70 beats min^−1^ while decreasing the risk of bradycardia (Table 1).

**Table 2:** Protocol for administration of investigational medicinal product, according to heart rate.

Heart rate(beats min^−1^)	Ivabradine or placebo(identical tablets)
<70	Omit
70–84	1 tablet (2.5 mg if ivabradine)
85–100	2 tablets (5 mg if ivabradine)
>100	3 tablets (7.5 mg if ivabradine)

### Blinding and procedures to minimise bias

This is a triple-blind trial, with trial participants, healthcare staff, investigators administering the treatment, and trial statistician all blinded to the treatment group allocation. To maintain blinding, placebo and ivabradine tablets are identical in shape, dimensions, and colour. Each IMP pack allocated to participants is labelled with a unique kit number to maintain blinding. The troponin measurement component of the primary outcome is analysed by individuals masked to the patient treatment allocation and clinical morbidity outcomes. Those assessing clinical outcomes (research associates and principal investigators) are not directly involved in the care of participants and will be unaware of treatment group allocation.

### Data collection

Postoperative outcomes will be recorded by research staff that are unaware of study group allocation on paper case report forms before electronic data entry using a secure web-based data entry platform. Data collection including participant characteristics before surgery is summarised in Supplementary material. Data will be collected from all participants randomised regardless of whether the participant received the intervention according to the trial protocol or not. The occurrence of a specified clinical outcome will be confirmed by the local principal investigator.

### Data monitoring

The sponsor will have oversight of trial conduct at sites, with the Trial Management Group having day-to-day responsibility for quality control and quality assurance of the data collected. An independent Data Monitoring and Ethics Committee (DMEC) and a Trial Steering Committee (TSC) review trial progress at least every 6 months, in accordance with an agreed charter. No formal interim analysis for efficacy is planned. The DMEC will monitor the safety and efficacy of the interventions during the period of recruitment into the trial, and also patient recruitment, data quality, protocol compliance, and loss to follow-up. The TSC will receive recommendations from the DMEC.

### Trial outcomes

#### Primary endpoint

The primary endpoint is a composite of myocardial injury associated with morbidity within 7 days of surgery. Myocardial injury is defined using the VISION study criteria,^3^ provided high-sensitivity troponin T (Elecsys; Roche Diagnostics; Rotkreuz, Switzerland) concentration ≥15 ng L^−1^ on day 1, 2, or 3 after surgery or increase of ≥5 ng L^−1^ from the preoperative value on day 1, 2, or 3 after surgery when the preoperative value is ≥15 ng L^−1^. The Elecsys Troponin T hs assay uses two monoclonal antibodies specifically directed against human cTnT, recognising two epitopes (amino acid positions 125–131, 136–147) located in the central part of the CTnT protein comprising 288 amino acids. Postoperative morbidity is systematically recorded using the Postoperative Morbidity Survey (POMS) on day 3 and day 7 after surgery.^30^

#### Secondary endpoints

The secondary endpoints include: (1) myocardial injury (as defined earlier); (2) morbidity 2–3 and 6–7 days after surgery, as defined by the POMS; (3) absolute concentrations of serum high-sensitivity troponin T measured on day 1, 2, and 3 after surgery; (4) mortality within 180 days from surgery; and (5) predefined complications at day 30 after surgery graded using the Clavien–Dindo classification.^31^

### Planned process measures

The planned process measures include (1) duration of hospital stay from surgery (number of calendar days from surgery until hospital discharge) and (2) number of calendar days requiring critical care admission (level 2 and 3) up to 30 days from surgery.

### Assessment of outcomes after hospital discharge

Participants discharged from hospital before day 30 will be contacted shortly after day 30 to establish any additional healthcare encounters since discharge, including hospital readmission. For participants who have received further treatment or seen a health professional since discharge, further details will be collected directly from the hospital/doctor or from the participant's health records. Mortality will be established by a participant medical record review, data from NHS Digital for participants in UK sites, or both. Morbidity outcomes will be assessed by a review of the participant's medical records, and by telephone interview in the same way as the primary outcome for 30-day outcomes.

### Protocol compliance monitoring

The tablets will be taken by the patient in the presence of a member of the direct clinical care team who will ensure that all required tables are swallowed. The number of tablets administered and the time of administration will be recorded on the electronic case report form (eCRF). If an incorrect number of tablets are administered or a dose is missed, a protocol deviation form will be completed.

### Safety and predefined adverse events

Safety data will be reported based on reviewing the patients' medical records and discussion with the patient. As ivabradine is already licenced and well-studied in the target age group, only certain safety events as outlined below will be collected as part of the trial procedures. Owing to the specificity of ivabradine on the sinoatrial node, only events specific to the drug action are recorded. It is expected that patients undergoing noncardiac surgery may often suffer from medical complications, up to and including death. As a result, a large number of participants will experience complications after surgery, which are completely unrelated to the trial intervention. These surgical complications will be recorded separately as part of outcome data.

Pharmacovigilance reporting will start from the completion of informed consent and stop no earlier than on day 30, the last day of talking to the patient. This will require either (1) review of the participant's medical notes if in hospital or (2) daily telephone call with the participant. In the event the research team is unable to speak with the participant, their general practitioner will be contacted. Although the events listed below are often observed in high-risk surgical patients, they will serve as safety outcome measures for the trial: (1) bradycardia <45 beats min^−1^ requiring rescue therapy according to local hospital guidelines, pacing detected as part of routine clinical care within the first 6 postoperative days, or both; (2) atrial fibrillation detected as part of routine clinical care within the first 6 postoperative days; (3) tachycardia >100 beats min^−1^ requiring treatment as recorded clinically within the first 6 postoperative days; (4) hypotension (MAP <60 mm Hg) detected as part of routine clinical care requiring pressor infusion within the first 6 days postoperative days; and (5) phosphenes (the experience of seeing light without light actually entering the eye) within the first 6 postoperative days.

### Sample size

The total sample size is 350 patients, with 175 participants assigned randomly to each arm. This will include a dropout rate of 1%. This sample size is based on VISION-UK data which showed that morbidity associated with troponin elevation (≥15 ng L^−1^) in the first 24 h after surgery is experienced by 59% of patients with similar characteristics.^32^ In contrast, 41% of patients without any troponin elevation sustained postoperative morbidity, representing a 31% lower relative risk of morbidity after surgery compared with patients who sustained myocardial injury defined by elevation in troponin. Assuming a conservative relative risk reduction of 25%, 173 patients will be required in each arm if ivabradine reduces the incidence of myocardial injury-associated morbidity from 59% (placebo) to 44% (α=0.05; 1–β=0.8).

### Statistical analysis

A full statistical analysis plan will be developed before any interim or final analysis and published online at https://www.qmul.ac.uk/ccpmg/sops--saps/statistical-analysis-plans-saps/. During the recruitment period, the trial statistician will perform a safety analysis if specifically requested by the DMEC, as outlined in the DMEC charter.

All analyses will be conducted according to the intention-to-treat principle, with all randomised participants with a recorded outcome analysed according to the treatment to which they were randomised. The primary outcome will be analysed using a logistic regression model. The model will be adjusted for minimisation variables (trial centre and planned surgical procedure [surgery involving the gut, all other surgery]). The model will also be adjusted for pre-specified baseline covariates entered into the model as fixed factors. Preoperative heart rate before the first IMP administration will be included as a continuous variable, assuming a linear association with the outcome. The magnitude of the treatment effect will be reported as an adjusted odds ratio (OR) with a 95% confidence interval (CI). Significance will be set at *P*<0.05. Similar analyses of secondary outcomes will be undertaken by intention-to-treat principle, according to the treatment to which they were randomised.

### Pre-specified subgroup analyses

A subgroup analysis will be performed for the primary outcome to assess whether the impact of targeted heart rate control differs in patients at the highest risk of myocardial injury: (1) preoperative heart rate ≥83 beats min^−1^,^12^^,^^13^ (2) raised preoperative NT-proBNP (>100 pg ml^−1^) which is prognostic for poorer perioperative outcomes,^33^^,^^34^ and (3) impaired orthostatic heart rate, an additional measure of cardiac vagal dysfunction.^29^ An additional mechanistic study in a subgroup of patients will explore autonomic changes after ivabradine or placebo dosing through the perioperative period.

### Patient and public involvement

The FUNNY trial was reviewed in detail Public Involvement and Engagement (PCPIE) in Research Group that was formed to provide high-quality guidance on research proposals in the field of perioperative medicine. Detailed feedback from this group has informed both the design and conduct of the trial. The group was informed on the design and implementation of PROTECTIN, the pilot study of ivabradine. The trial meets several priorities in the 10 most important research questions published by the James Lind Alliance Priority Setting Partnership for Anaesthesia and Perioperative Care. A previous Royal College of Anaesthetists PCPIE lead joined the FUNNY project group as a lay representative. This member has been involved throughout the preparation of the original grant, trial, and provides detailed input into issues of safety and the experience of participating patients. A lay summary of the trial results will be available to participants.

### Ethics and dissemination

The FUNNY trial has been approved by the UK National Research Ethics Service and ethics committees in all participating countries. All participating centres have full ethical approval. A detailed scientific report will be submitted to a widely accessible scientific journal, with further dissemination including presentations at international scientific meetings, public presentations, webcasts, and to professional organisations, front-line healthcare workers, patients, and the public. Deidentified data will also be shared with other authenticated researchers for further research in a timely and responsible manner, provided confidentiality of the information is preserved. Requests for data sharing will be considered by the Chief Investigator (CI) in accordance with the Queen Mary University of London data sharing policy.

## Discussion

This will be the first phase 2a randomised trial examining the effectiveness of personalised, targeted heart rate control in patients at risk of myocardial injury and associated complications who are undergoing major elective noncardiac surgery. The pharmacological action of ivabradine and characteristics pf preoperative physiology will provide mechanistic insight into potential different aetiologies of postoperative myocardial injury.

The primary outcome is morbidity associated with myocardial injury, defined by the POMS and elevations in high-sensitivity troponin T, respectively. POMS provides a comprehensive assessment of the day-to-day health status of patients after surgery, and captures patient-centred outcomes that are repeated for longitudinal assessment. Our previous work showed that an early elevation in circulating troponin within 12 h of elective noncardiac surgery precedes the subsequent development of POMS-assessed noncardiac organ dysfunction.^32^ Patients with elevated troponin within 12 h of surgery had a higher risk of proven/suspected infectious morbidity (OR 1.54; 95% CI 1.24–1.91) and critical care utilisation (OR 2.05; 95% CI 1.73–2.43). Clavien–Dindo grade ≥3 complications occurred in 167/992 (16.8%) patients with elevated troponin within 12 h of surgery, compared with 319/3343 (9.5%) patients with hsTnT <99th centile (OR 1.78; 95% CI 1.48–2.14). If myocardial injury is mechanistically linked to noncardiac complications, reducing its incidence should drive a parallel benefit in fewer infections, renal injury, and other common, low-grade morbidity that keep patients in hospital for longer.

The precedent for cardiac tissue injury fuelling distant organ dysfunction (and hence morbidity) is well established in experimental models of immunosuppression after tissue injury. Murine cardiac dysfunction after experimental myocardial infarction increases susceptibility to bacterial infection owing to impaired phagocytosis in hepatic macrophages.^35^ Lymphopaenia is a prototypical response after tissue injury, triggered by a hyperacute pro-inflammatory response after disparate sites of organ injury.^36^ Cell-free DNA released by tissue injury binds to the inflammasome activator absent in melanoma 2 (AIM2) in myeloid cells.^37^ In turn, AIM2 inflammasome-dependent interleukin-1β secretion by monocytes leads to extrinsic T-cell apoptosis. In support of these observations, clinical myocardial injury and complications are independently associated with a lower absolute preoperative lymphocyte count^38^ and a higher absolute preoperative monocyte count.^5^

In the general population, lower resting heart rates are protective against cardiovascular pathology.^39^ Heart rate is a key determinant of the balance between myocardial oxygen supply and demand.^40^ However, lowering the heart rate before noncardiac surgery using negative chronotropic agents such as beta-blockers shows that off-target effects of reducing oxygen demand can be counterproductive.^14^ It is likely that fixed doses of heart rate-lowering therapy that are not titrated to a target heart rate threshold are detrimental through hypoperfusion after profound hypotension.^14^ Although preoperative heart reduction may therefore be beneficial, excessive reductions therefore appear very likely to be harmful.^41^ Moreover, if elevated perioperative heart rate serves merely as a marker of vagal autonomic impairment, a strategy of reducing heart rate may actually be harmful.

Experimental work using *in vitro* endothelial models shows that mimicking the physiological frequencies of higher heart rate results in a proinflammatory transcriptomic response.^42^ Thus, limiting laminar shear stress is likely to protect endothelial function and reduce systemic inflammation.^43^, ^44^, ^45^ Chronic heart rate reduction by ivabradine prevents cardiac, renovascular, and cerebrovascular endothelial dysfunction in a genetic model of dyslipidaemia in mice.^46^ Administration of intravenous ivabradine 24 h after sepsis as a result of caecal ligation and puncture restored microvascular function towards baseline values.^47^

As higher preoperative heart rates indicate cardiac vagal impairment, it is likely that relative tachycardia represents generalised parasympathetic impairment. Direct and indirect parasympathetically mediated mechanisms that confer extracardiac, multiorgan protection are likely to be involved in minimising morbidity that follows significant tissue trauma associated with major noncardiac surgery.^41^ Both pre-existing^22^ and acquired^23^ loss of vagal activity are mechanistically associated with exacerbation of systemic inflammation,^48^ which in turn promotes or worsens myocardial injury,^18^ cardiac arrythmias,^17^ and lung injury.^20^ Experimental data also suggest that a parasympathetic–renal axis contributes to acute kidney injury, by reducing the inflammatory response to renal ischaemia–reperfusion injury.^49^ The parasympathetic neurotransmitters acetylcholine and vasoactive intestinal peptide (VIP) confer organ protection through direct and indirect mechanisms. For example, acetylcholine via the α7 nicotinic acetylcholine receptor of macrophages reduces systemic inflammation.^21^ Preservation of adequate coronary artery blood flow during cardiovascular stress (e.g. exercise) is mediated by VIP.^50^

The strengths of this phase 2 RCT are that patients, investigators, and clinical teams will be blinded through the use of placebo tablets. Participants and clinicians will be blinded to both treatment and the troponin results. Heart rate changes during the intervention period (surgery day to postoperative day 2) are dynamic and influenced by multiple factors. Non-IMP interventions remain unrestricted. In our prior pilot trial with the same heart rate-guided ivabradine or placebo intervention, clinical staff identifying which trial arm patients were allocated did not emerge as an operational issue for the execution of FUNNY.^26^ The multicentre, international design and broad inclusion criteria are generalisable to a larger number of patients undergoing noncardiac surgery and therefore address a major healthcare burden of clear importance to patients.

In summary, this phase 2b study will establish whether targeted heart rate control reduces morbidity after surgery associated with early myocardial injury, using ivabradine to selectively slow the heart rate without impairing perioperative haemodynamic control.

## Authors' contributions

Study protocol design: GLA

Manuscript drafting of paper: all authors

Subsequent revision of manuscript: all authors

## Funding

National Institute for Health and Care Research Advanced Fellowship (NIHR 300097 to GLA); British Heart Foundation programme (RG/19/5/34463 to GLA).

## Declarations of interests

GLA is Editor of the *British Journal of Anaesthesia*, has UK patent application 2011523.4 (Neuromodulation for the treatment of critical illness). The other authors declare that they have no conflicts of interest.

## Appendix B

FUNNY study investigators: Bernardo Bollen Pinto, Benjamin Shelley, Priyanthi Dias, Salma Begum, Florence Ennahdi-Elidrissi, Russell Hewson, Anna Wozniak, Shaun M. May, Mareena Joseph, Agustine Miguel Saavedra, Tim Martin, Onika Ottley, Ana Santos, Fatima Seidu, Stéphanie Mulin, Stéphane Luise, Isabelle Pichon, John Daniels, Béatrice Gil-Wey, Soraya Bicher, Gaël Rais, Christene Aitken, Elizabeth Boyd, Patricia Griffen, Charlene Hamilton, Kathryn Valdeavella, Rhiannon McAreavey, Phillip McCall, Alfie Lloyd, Jocelyn Barr, Julie Buckley, Anne Marie Tiah, Henrike Janssen, Lisa Kandala, Angela Fitzpatrick, Alexander Lysomirski, Ahmed Ahltobi, Ana Gutierrez del Arroyo, Tom E. F. Abbott, Akshaykumar Patel, Kamran Khan, Rupert M. Pearse, Gareth L. Ackland
