# Supplementary material for: Targeted heart rate control using the funny current inhibitor ivabradine to reduce morbidity in patients undergoing noncardiac surgery: study protocol for a phase 2a, triple-blind, placebo-controlled randomised trial
Source: BJA Open. 2025 Feb 19;13:100378. doi: 10.1016/j.bjao.2025.100378 (PMC11889562; doi:10.1016/j.bjao.2025.100378)
Supplement: Multimedia component 1 [file mmc1.pdf]

Targeted heart rate control using the funny current inhibitor ivabradine to reduce morbidity in patients undergoing non-cardiac surgery: study protocol for a phase IIa, triple blind, placebo controlled randomised trial.

SUPPLEMENTARY DATA

Contents

Patient information sheet 2

Data collection 9

Queen Mary University of London

NHS Barts Health NHS Trust

funny

Does ivabradine reduce heart injury and help patients recover quicker after surgery by slowing the heart rate?

PATIENT INFORMATION SHEET (UK)
Version 3.0 21.04.2021
Principal Investigator: Professor Gareth Ackland
IRAS: 1003561

Introduction

We are a research team from the Queen Mary University of London (QMUL) working with doctors and nurses at Royal London Hospital. We are inviting you to take part in a clinical research study, which we hope will improve the care of patients who undergo major surgery. Before you decide, it is important to understand why we are doing this research and what it involves. Please take your time to read the following information and decide whether or not you wish to take part. Talk to your family and friends about the study if you wish. Ask us if anything is unclear.

Why are we doing this research?

We are studying alternative ways of looking after surgical patients and we hope this treatment will improve patient care. Previous studies have shown that patients with higher heart rates before, during and after surgery are more vulnerable to developing complications post-operatively. We are investigating whether reducing heart rate by using a drug called ivabradine will protect it from damage and reduce postoperative complications, ensuring that patients can return home sooner and in better health.

Why have I been invited?

We have invited you because your surgeon has recommended you for an operation where this treatment may have particular benefit, and you have been identified as being at higher risk of morbidity following the surgery.

Do I have to take part?

No. It is up to you to decide whether or not to take part in the study. If you decide to take part, we will ask you to sign a consent form.

What will happen to me if I take part?

Study drug/placebo

Sometimes we do not know which way of treating patients is best. To find out, we need to put people into groups that receive different treatments. The results from the groups are then compared to see if one is better than the other. To try to ensure the groups are the same to start with, you will be randomly allocated (like flipping a coin). In this study 50% of participants will receive the study drug (ivabradine) and 50% will receive a placebo ('dummy' treatment). Neither you nor your doctor will be able to decide which study treatment you receive. Keeping everything exactly the same apart from what is contained in the tablet allows us to study just the effects of ivabradine. The study is blinded, meaning that neither you nor your doctor will know which treatment group you are in (although if your doctor needs to find out they can do so). Unfortunately you will not be able to find out which treatment you received until the study has finished. Regardless of which group you are in, you will be asked to take either 0, 1, 2 or 3 tablets twice a day from the day of your operation until day two after your operation (unless you are discharged earlier). The number of tablets you will be asked to take will depend on your heart rate within the previous hour so the number of tablets may vary between doses. You will only be asked to take the tablets whilst you are in hospital, you will not take any tablets home with you.

Heart rate measurement before surgery

Before your operation, the research team will measure your heart rate by conducting a simple sitting to standing test. For this test you will be asked to sit for 3 minutes before standing up for a further 3 minutes whilst wearing a heart rate monitor.

Blood samples

The research team at your hospital will also obtain a blood sample (approximately 3 teaspoons) before surgery and on the first three days after surgery (whenever possible, this will be done at the same time as your routinely collected blood samples) to check whether your heart shows signs of stress. If you agree, the blood samples obtained during the course of this study will also be used for closely related future research studies. The samples will be stored for five years before being destroyed.

Continuous heart rate and blood pressure measurements

Depending on the hospital, you may be asked to wear a Holter monitor, which continuously records your heart rate and a blood pressure (BP) cuff which continuously records your blood pressure. The recordings will start on the day of your operation and last up to three days after surgery or hospital discharge, whichever comes first. Throughout your hospital stay, the local research team will come to see you to follow your recovery and review your medical notes.

Follow-up

If you are discharged from hospital within three days from your last dose, the research team will contact you daily to ask about your wellbeing. They will do so until three days have elapsed from your last dose. If they cannot get hold of you, they will contact your General Practitioner (GP). If you have any concerns about your health following the hospital discharge, please get in touch with the research team via the contact details at the bottom of this information sheet. One month after your surgery a member of the research team at your hospital will contact you by telephone to see how you are doing and ask some brief questions about your recovery. This telephone conversation will take less than five minutes. We may also contact your GP to gather basic information about your recovery one month and six months after your surgery.

What are the possible risks and benefits of taking part?

The treatment we are investigating is generally safe and you could benefit from the intervention but we can't guarantee this. The information we get from this study may benefit people undergoing surgery in the future. Ivabradine has been on the EU market for over 15 years and is commonly used to treat chronic heart failure and the symptoms of chronic angina (chest pain). Your doctors at the hospital are aware that you are on this study and you will be closely monitored throughout your hospital stay. If necessary, adjustments to your treatment will be made to make sure you are safe. We will also let your GP know of your participation in the study so that s/he is aware that you might have received ivabradine during your hospital stay.

What are the possible side effects?

Ivabradine is generally safe and most people do not have any problems but like all medicines, it can cause side effects in some people. The side effects of ivabradine include:

- *Very common (may affect more than 1 in 10 people):* Luminous visual phenomena (brief moments of increased brightness, most often caused by sudden changes in light intensity). They can also be described as a halo, coloured flashes, image decomposition or multiple

images. They generally occur within the first two months of treatment after which they may occur repeatedly and resolve during or after treatment.

- *Common (may affect up to 1 in 10 people):* Modification in the heart functioning (the symptoms are a slowing down of the heart rate). They particularly occur within the first 2 to 3 months of treatment initiation. Other common side effects include headache, dizziness, blurred vision (cloudy vision), irregular rapid contraction of the heart, abnormal perception of heartbeat and uncontrolled blood pressure.
- *Uncommon (may affect up to 1 in 100 people):* Fainting, double vision, impaired vision, spinning sensation (vertigo), palpitations and cardiac extra beats, low blood pressure, difficulty breathing (dyspnoea), feeling sick (nausea), constipation, diarrhoea, abdominal pain, skin rash, muscle spasms, feeling of tiredness, feeling of weakness, swollen face, tongue or throat, difficulty in breathing or swallowing (angioedema), changes in laboratory parameters: an excess of eosinophils (a type of white blood cell), high blood levels of uric acid, elevated creatinine in blood (a breakdown product of muscle), abnormal ECG heart tracing.
- *Rare (may affect up to 1 in 1,000 people):* Skin reddening, itching, urticaria, feeling unwell.
- *Very rare (may affect up to 1 in 10,000 people):* Irregular heartbeats.

What will happen if I don't want to carry on with the study?

If you decide not to take part, or later to withdraw, this will not affect the standard of care you receive. You are free to stop taking part at any time, without giving a reason but the research team will keep your research data and the blood samples that have already been collected. You can find out what would happen with your data before you agree to take part in a study. If you choose to stop taking part in the study, we would like to continue collecting information about your recovery from your medical and GP records. If you do not want this to happen, tell us and we will stop. For safety reasons, we are required by the Medicines and Healthcare products Regulatory Agency (MHRA) to follow your recovery for three days since the last dose of the study drug.

What if I am not happy about the study?

Taking part in the study does not affect the way you are cared for in hospital. However, if you have a concern about any aspect of this study, you should ask to speak with someone from the research team at the hospital, who will do their best to answer your questions. You can also contact them on the telephone number at the bottom of this information sheet. You may also contact your Patient Advisory Liaison Service (PALS) if you have any concerns regarding the care you have received, or as an initial point of contact if you have a complaint. Please contact them via telephone 0203

594 2040 or email BHNT.CentralComplaints@nhs.net. You can also visit by asking at hospital reception. QMUL has agreed that if you are harmed as a result of your participation in the study, you will be compensated, provided that, on the balance of probabilities, an injury was caused as a direct result of the procedures you received during the course of the study. These special compensation arrangements apply where an injury is caused to you that would not have occurred if you were not in the study. These arrangements do not affect your right to pursue a claim through legal action.

How will we use information about you?

Authorised members of the research team at your hospital will need to access information from your medical records so that they can collect the information required for this research project. This information will include your initials only. Information about your medical status may also be requested from your GP. People who do not need to know who you are will not be able to see your name or contact details. Your data will have a code number instead. We will keep all information about you safe and secure. Our procedures for handling, processing, storage and destruction of data are compliant with the General Data Protection Regulation Guidelines 2018 and Data Protection Act 2018.

You can find more information on how researchers use information from patients on https://www.hra.nhs.uk/planning-and-improving-research/policies-standards-legislation/data-protection-and-information-governance/gdpr-guidance/templates/template-wording-for-generic-information-document/. If you would like to receive a paper copy of this information, please ask the research team at your hospital.

Where can you find out more about how your information is used?

You can find out more about how your information is used:

- at http://www.jrmo.org.uk/ or by contacting the QMUL data protection officer: Jonathan Morgan data-protection@qmul.ac.uk
- by contacting the trial coordinating team on admin@funnytrial.org
- by ringing us on +44 (0)20 3594 0352

What are your choices about how your information is used?

We need to manage your records in specific ways for the research to be reliable. This means that we won't be able to let you see or change the data we hold about you. This is because research could go wrong if data is removed or changed.

Who is organising and funding the research?

The study is funded by the National Institute for Health Research. The study is run by the Critical Care and Perioperative Medicine Research Group at QMUL. QMUL will also act as the Sponsor and the data controller for this study. Neither you nor your medical staff will be paid for your participation in this study.

Who has reviewed the study?

All research in the NHS is reviewed by an independent Research Ethics Committee (REC), to protect the interests of the patients who take part. This study has been reviewed and granted a favourable opinion by the NHS Research Ethics committee and has also been approved by the Health Research Authority, MHRA and the NHS Research and Development department in your hospital.

What will happen to the results of this study?

Once we have finished the study, we will keep some of the data so we can check the results. We hope to publish the results in a scientific journal. However, we will write our reports in a way that no-one can work out that you took part in the study. Copies of the final scientific report will be available on request. We are required by research regulations to keep the study data for a minimum of 25 years after the study has been completed in case we need to check it. The data will be kept in a secure facility only accessible to authorised personnel. The researchers from your hospital will only share your initials with QMUL.

Thank you!

Thank you for considering taking part in this study and for reading this information sheet, which is yours to keep. If you decide to take part in the study, you will also be given a copy of your signed consent form.

Your study doctor is:	Your research/ specialist nurse is:
Name: Professor Gareth Ackland	Name:
Contact phone number:	Contact phone number:

Data collection

Randomisation data

- Initials
- Date of consent and surgery
- Age
- Gender
- Checklist to ensure the patient meets the eligibility criteria
- Planned surgical procedure category (surgery involving the gut, all other surgery)
- Trial ID (generated automatically at the point of randomisation)

Baseline data

- Orthostatic test (see section 12.1)
- Co-morbidities
- Smoking status (within the last 14 days)
- Cardiovascular medications
- Height
- Weight
- Ethnicity (to calculate estimated glomerular filtration rate)
- Laboratory values (haemoglobin, creatinine, neutrophil count, lymphocyte count, albumin)
- Pre-operative resting heart rate and rhythm one hour before IMP administration
- Number of IMP tablets administered before surgery
- Time of IMP administration
- Continuous heart rate (measured with Holter monitor/telemetry)[1]
- Continuous blood pressure (measured with ABP/telemetry)[1]
- Planned level of care on the first night after surgery
- AE/SAE review

[1]selected sites only

During surgery

- Start and end times of surgery
- Surgical procedure category (surgery involving the gut, all other surgery)

- Surgical technique (open, laparoscopic or laparoscopic assisted, laparoscopic converted to open)
- Anaesthetic technique (general anaesthesia, epidural, spinal, other regional anaesthesia, sedation)
- Endotracheal intubation for surgery
- Arrhythmias
- Volume of blood products administered (packed red cells, all other products)
- Continuous heart rate (measured with Holter monitor/telemetry)[1]
- Continuous blood pressure (measured with ABP/telemetry)[1]
- Systolic blood pressure <90mmHg (Y/N, lowest value, duration, IV fluids, pressor treatment [e.g. phenylephrine, ephedrine, metaraminol, norepinephrine])
- Heart rate >100bpm (Y/N, highest value, duration, IV fluids, drug treatment)
- AE/SAE review

[1]selected sites only

Postoperative period

- Arrhythmias
- Creatinine measurement
- Volume of blood products administered (packed red cells, all other products)
- Urine output
- Systolic blood pressure <90mmHg (Y/N, lowest value, duration, IV fluids, pressor treatment [e.g. phenylephrine, ephedrine, metaraminol, norepinephrine])
- Heart rate >100bpm (Y/N, highest value, duration, IV fluids, drug treatment)
- Rescue treatment for bradycardia since the previous dose
- Resting heart rate and rhythm one hour before IMP administration
- Number of IMP tablets administered
- Time of IMP administration
- Actual level of care on the first night after surgery
- Continuous heart rate (measured with Holter monitor/telemetry)[1]
- Continuous blood pressure (measured with ABP/telemetry)[1]
- AE/SAE review

[1]selected sites only

Postoperative day one

- Arrhythmias

- Volume of blood products administered (packed red cells, all other products)
- Urine output
- Blood sample
- Cardiovascular medications
- Level of care on the second night after surgery
- Systolic blood pressure <90mmHg (Y/N, lowest value, duration, IV fluids, pressor treatment [e.g. phenylephrine, ephedrine, metaraminol, norepinephrine])
- Heart rate >100bpm (Y/N, highest value, duration, IV fluids, drug treatment)
- Laboratory values (haemoglobin, creatinine, neutrophil count, lymphocyte count, albumin)
- Rescue treatment for bradycardia since the previous dose[2]
- Resting heart rate and rhythm one hour before IMP administration[2]
- Number of IMP tablets administered[2]
- Time of IMP administration[2]
- Continuous heart rate (measured with Holter monitor/telemetry)[1]
- Continuous blood pressure (measured with ABP/telemetry)[1]
- AE/SAE review
- Hospital discharge on postoperative day one

[1]selected sites only

[2]recorded twice daily

Postoperative day two

- Arrhythmias
- Volume of blood products administered (packed red cells, all other products)
- Urine output
- Blood sample
- Cardiovascular medications
- Systolic blood pressure <90mmHg (Y/N, lowest value, duration, IV fluids, pressor treatment [e.g. phenylephrine, ephedrine, metaraminol, norepinephrine])
- Heart rate >100bpm (Y/N, highest value, duration, IV fluids, drug treatment)
- Laboratory values (haemoglobin, creatinine, neutrophil count, lymphocyte count, albumin)
- Rescue treatment for bradycardia since the previous dose[2]
- Resting heart rate and rhythm one hour before IMP administration[2]
- Number of IMP tablets administered[2]

- Time of IMP administration[2]
- Continuous heart rate (measured with Holter monitor/telemetry)[1]
- Continuous blood pressure (measured with ABP/telemetry)[1]
- AE/SAE review
- Hospital discharge on postoperative day two

[1]selected sites only

[2]recorded twice daily

Postoperative day three

- Arrhythmias
- Volume of blood products administered (packed red cells, all other products)
- Urine output
- Blood sample
- Cardiovascular medications
- Systolic blood pressure <90mmHg (Y/N, lowest value, duration, IV fluids, pressor treatment [e.g. phenylephrine, ephedrine, metaraminol, norepinephrine])
- Heart rate >100bpm (Y/N, highest value, duration, IV fluids, drug treatment)
- Laboratory values (haemoglobin, creatinine, neutrophil count, lymphocyte count, albumin)
- Continuous heart rate (measured with Holter monitor/telemetry)[1]
- Continuous blood pressure (measured with ABP/telemetry)[1]
- AE/SAE review
- Hospital discharge on postoperative day three
- POMS (see appendix B)

[1]selected sites only

[2]recorded twice daily

Postoperative day four to five

- AE/SAE review

Postoperative day seven

- POMS (see appendix B)
- AE/SAE review

Hospital discharge

- Blood pressure (last three measurements before discharge)
- Heart rate (last three measurements before discharge)
- AE/SAE review

Follow-up data

30-day follow-up

- Date of follow-up
- Mortality status within 30 days of surgery
- Clavien-Dindo graded cardiac complications within 30 days of surgery
- Clavien-Dindo graded respiratory complications within 30 days of surgery
- Clavien-Dindo graded infective complications within 30 days of surgery
- Other predefined Clavien-Dindo graded complications within 30 days of surgery
- Acute kidney injury (using KDIGO staging criteria)
- Additional treatments
- Patients admitted to a critical care unit
- Number of days in level two and level three critical care within 30 days of surgery
- Duration of primary hospital admission
- Re-admission to hospital within 30 days of surgery
- Blinding status
- AE/SAE review

180-day follow-up

- Date of follow-up
- Mortality status within 180 days of surgery

Supplementary forms:

- Withdrawal
- AE/SAE
- Protocol deviation
    - Incorrect dose given
    - Missed dose
    - Other deviation
